# Structural elucidation and antidiabetic activity of polysaccharides from the parasitic plant *Orobanche cumana*

**DOI:** 10.3389/fnut.2026.1785297

**Published:** 2026-04-13

**Authors:** Shixiong Yang, Hengli Shen, Xichen Qiao, Qiang Song, Xizun Tan, Xiaoyu Li, Pengyue Guo, You Zhang, Hairong Xiong, Yao Wu, Zongbao Zhou

**Affiliations:** 1 College of Life Science, South-Central Minzu University, Wuhan, China; 2 Central Laboratory, Xiaogan Hospital Affiliated to Wuhan University of Science and Technology, Xiaogan, China; 3 Hubei Key Laboratory of Resource Utilization and Quality Control of Characteristic Crops, College of Life Science and Technology, Hubei Engineering University, Xiaogan, China

**Keywords:** antidiabetic effect, antioxidant activity, *Orobanche cumana*, plant polysaccharides, sequential extraction

## Abstract

**Introduction:**

This study systematically evaluated the therapeutic potential of polysaccharides from the agricultural plant *Orobanche cumana* for diabetes management.

**Methods:**

Three polysaccharide fractions (OCP-1, OCP-2, OCP-3) with distinct structural profiles were obtained through sequential extraction using water at room temperature, high-temperature water, and alkaline solution, respectively.

**Results:**

Among them, the alkaline-extracted OCP-3 exhibited the most favorable properties, which was characterized as a rhamnogalacturonan-I-rich polysaccharide with a low molecular weight of 66,273 Da. OCP-3 demonstrated strong antioxidant activity by effectively scavenging 2,2-diphenyl-1-picrylhydrazyl, hydroxyl, and superoxide anion radicals, and significantly inhibited both *α*-amylase (IC₅₀ = 98.5 μg/mL) and α-glucosidase (IC₅₀ = 56.1 μg/mL). In streptozotocin-induced type 2 diabetic male C57BL/6 mice, OCP-3 treatment alleviated hyperglycemic symptoms, improved insulin sensitivity, and provided substantial protection against pancreatic, hepatic, and renal damage. Specifically, OCP-3 reduced fasting blood glucose from 31.3 to 17.2 mM and restored insulin levels to 10.34 mU/L, and improved oral glucose tolerance (AUC = 39.83 h·mmol/L), while maintaining an excellent safety profile in toxicological assessment.

**Discussion:**

These findings not only validate the traditional use of *Orobanche cumana* but also establish OCP-3 as a safe and effective candidate for diabetes treatment, offering a sustainable approach to valorizing this agricultural plant.

## 1 Introduction

Sunflower broomrape (*Orobanche cumana Wallr.*) is a holoparasitic plant that poses a major threat to global sunflower production, causing yield losses exceeding 50% in infested fields (1). This root parasite forms specialized haustorial connections with host roots to extract water and nutrients, completing its life cycle entirely at the host’s expense. The weed’s subterranean development, rapid evolution of resistance, and efficient seed dispersal have established it as a persistent challenge to sustainable sunflower cultivation (2, 3). Paradoxically, despite its agricultural detriment, *O. cumana* possesses significant therapeutic potential recognized in traditional Uyghur medicine, where it has been used to treat kidney yang deficiency, sexual dysfunction, and musculoskeletal disorders (4, 5). Contemporary pharmacological studies have corroborated its diverse bioactivities, including anti-inflammatory, antioxidant, and antimicrobial effects (6). Notably, historical Uyghur medical records document its use in managing hyperglycemia, and recent *in vivo* animal studies have shown that extracts of *O. cumana* can reduce blood glucose and lipid levels in diabetic mice (7). Nonetheless, traditional preparation methods—mainly water-based decoctions—are not standardized, and the precise bioactive constituents responsible for these effects remain poorly characterized, impeding further development into modern therapeutics.

Diabetes mellitus affects over 700 million people worldwide, driving demand for safer therapeutic alternatives to conventional drugs, which are often limited by gastrointestinal side effects and hypoglycemia risk (8). Plant-derived heteropolysaccharides have emerged as promising alternatives due to their capacity to modulate multiple pathological pathways with minimal toxicity (9). Sun et al. isolated a novel *β*-1,3-mannan-rich polysaccharide (NAP-3, 428 kDa) from *Cistanche deserticola*, which exhibited strong inhibitory activity against *α*-glucosidase and α-amylase, with IC₅₀ values similar to acarbose (10). The isolation of a galactomannoglucan-type pectic polysaccharide from date palm heartwood, characterized by significant uronic acid content, demonstrated promising antidiabetic potential through a mixed-type inhibition of α-amylase and significant hypoglycemic effects in diabetic rats (11). These findings collectively highlight the promise of natural polysaccharides in diabetes therapy. Given the documented anti-hyperglycemic effects of *O. cumana* extracts and the established efficacy of plant polysaccharides in diabetes treatment, it is hypothesized that polysaccharide components within this parasitic plant may contribute significantly to its therapeutic properties. Nevertheless, systematic investigations characterizing *O. cumana* polysaccharides and evaluating their antidiabetic potential remain absent from current literature.

The present study was designed to systematically investigate the polysaccharide components of *O. cumana* as potential antidiabetic agents through an integrated experimental approach. The research strategy involved sequential extraction under varied conditions—room temperature, high temperature, and alkaline solution—to obtain three distinct polysaccharide fractions. These fractions then underwent comprehensive structural characterization to determine their chemical composition, molecular weight, monosaccharide profiles, and structural features. Subsequent evaluation included assessment of antioxidant capacities against multiple free radicals and inhibitory effects on key carbohydrate-digesting enzymes. The most promising fraction was selected for further investigation of its antidiabetic efficacy and safety profile using a streptozotocin-induced diabetic mouse model. This systematic investigation from structural characterization to multi-level bioactivity assessment aims to establish fundamental structure–activity relationships for *O. cumana* polysaccharides while exploring the potential of transforming this agricultural pest into a valuable therapeutic resource for diabetes management.

## 2 Materials and methods

### 2.1 Materials

Dried whole plants of *O. cumana* were harvested from the Inner Mongolia Autonomous Region, China, during the flowering season in July 2023, and were authenticated by a botanical taxonomist from College of Life Science and Technology, Hubei Engineering University. A voucher specimen (No. OC-202307) has been deposited in the Herbarium of Hubei Engineering University. Ethanol, sodium carbonate, and trifluoroacetic acid (TFA, ≥99.5%) were supplied by Shanghai Macklin Biochemical Co., Ltd. Monosaccharide standards (purity ≥98%) were obtained from the same supplier. All biochemical assay kits for antioxidant capacity and enzyme inhibition studies were provided by Solarbio Life Sciences (Beijing, China). Streptozotocin (STZ) and metformin hydrochloride were sourced from Sigma-Aldrich. The High-Fat Diet was purchased from Research Diets Inc. Cell culture reagents including DMEM and bovine serum albumin (BSA) were acquired from Gibco. AB-8 macroporous resin and dialysis membranes (7 kDa MWCO) were obtained from Tianjin Guangfu Fine Chemical Research Institute and Solarbio, respectively. Experimental animals were supplied by Wuhan Shulaibao Biotechnology Co., Ltd. All other chemicals used were of analytical grade.

### 2.2 Extraction procedures of polysaccharides from sunflower broomrape

**Figure 1:**
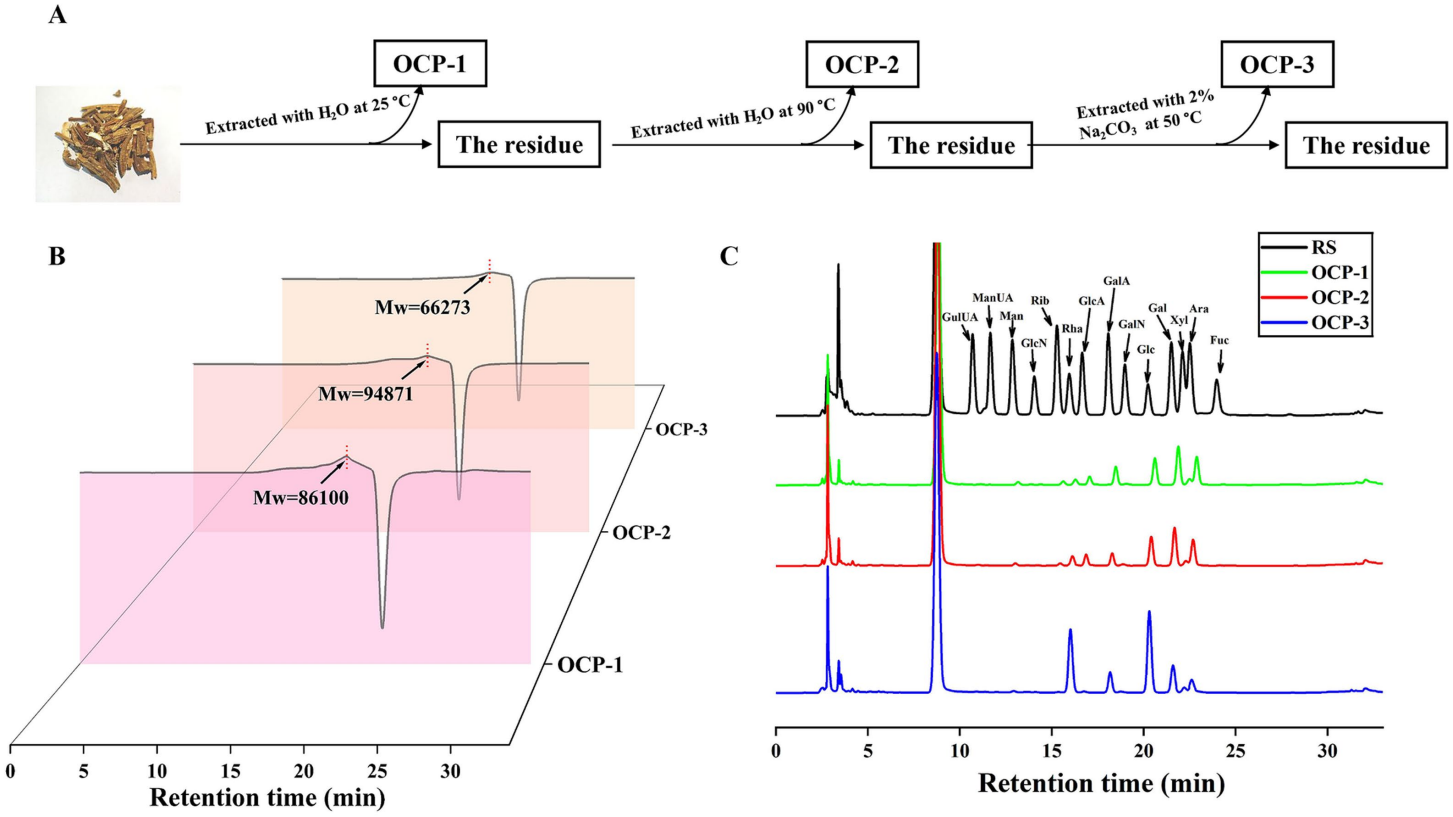
Extraction, molecular weight distribution, and monosaccharide composition of OCPs. **(A)** Schematic diagram of the sequential extraction procedure for OCP-1, OCP-2, and OCP-3. **(B)** The molecular weight distribution of OCPs (*n* = 3 independent experiments). **(C)** Monosaccharide composition analysis of OCPs (data presented as mean mol%, *n* = 3).

The polysaccharides from sunflower broomrape, *O. cumana* were sequentially extracted using a stepwise scheme designed to isolate fractions with distinct solubility profiles, as summarized in Figure 1A.

#### 2.2.1 Pretreatment and defatting

Two hundred gram dried powder of *O. cumana* was refluxed with 80% ethanol at a solid-to-liquid ratio of 1:10 (w/v) at 80 °C for 4 h. This pretreatment effectively removed lipids, pigments, and low-molecular-weight compounds. The resulting residue was collected by filtration and air-dried for further use.

#### 2.2.2 Room temperature extraction

The defatted powder was extracted with 5 L distilled water under continuous stirring at 25 °C for 2 h. This process was repeated three times. The combined filtrates were concentrated under reduced pressure, and polysaccharides were precipitated by adding ethanol to a final concentration of 80% (v/v), followed by incubation at 4 °C overnight. The precipitate was recovered by centrifugation, redissolved in distilled water, and purified using AB-8 macroporous resin to remove impurities and oligosaccharides. The resulting solution was dialyzed for 48 h and lyophilized to afford the polysaccharide fraction extracted at room temperature, designated OCP-1.

#### 2.2.3 High-temperature water extraction

The residue from the room temperature extraction step was further extracted with distilled water at 80 °C for 2 h, repeated three times. The combined filtrates were concentrated and precipitated with 80% ethanol. After centrifugation, the precipitate was redissolved, purified over AB-8 resin, dialyzed, and lyophilized as above, yielding the high-temperature water-soluble polysaccharide fraction, designated OCP-2.

#### 2.2.4 Alkaline extraction

The remaining residue was treated with 5 L 2% (w/v) sodium carbonate solution at 60 °C for 2 h, also in triplicate. The filtrates were combined, neutralized with dilute acetic acid, concentrated, and precipitated with 80% ethanol. The collected precipitate was redissolved, purified via AB-8 resin, dialyzed against distilled water, and lyophilized to give the alkaline-soluble polysaccharide, designated OCP-3.

#### 2.2.5 Chemical composition of OCPs

The chemical composition of OCPs was characterized using established methods. The extraction yield was calculated as the percentage of the dry weight of extracted OCPs relative to the initial dry weight of pretreated material. The total sugar content was determined by the phenol-sulfuric acid method using glucose as a standard, with absorbance measured at 485 nm (12). Residual protein content was determined according to Bradford’s method using BSA as standard, with absorbance read at 595 nm (13). Total phenolic content was measured using the Folin–Ciocalteu assay with gallic acid as standard, monitoring absorbance at 760 nm, and total flavonoid content was assessed by the Al (NO3)3 colorimetric method with catechin as standard, measuring absorbance at 510 nm (14). All analyses were performed in triplicate with appropriate standard curves, and results were expressed as mean values with standard deviations.

### 2.3 Structural properties of OCPs

#### 2.3.1 Molecular weight distribution

The molecular weights and homogeneity of OCPs were determined by high-performance gel permeation chromatography (HPGPC). The analysis was performed at 35 °C with ultra-pure water as the mobile phase at a flow rate of 0.5 mL/min. The samples (0.2 mg/mL) were filtered through 0.22 μm membranes prior to injection. A series of dextran standards with known molecular weights were used for calibration.

#### 2.3.2 Monosaccharide composition analysis

Monosaccharide constituents were identified using ion chromatography coupled with pulsed amperometric detection (15). Sample preparation involved hydrolyzing 10 mg of OCPs with 3 M trifluoroacetic acid at 115 °C for 8 h in sealed glass tubes. The hydrolysate was then evaporated to complete dryness under a nitrogen stream. Pre-column purification was performed using solid-phase extraction cartridges prior to instrumental analysis. The analytical separation employed a gradient elution program with 0.2 M NaOH and 1 M CH3COONa solutions at a constant flow rate of 0.8 mL/min. Column temperature was maintained at 30 °C throughout the analysis. Quantification was accomplished by constructing calibration curves from standard monosaccharide solutions covering the concentration range of 0.1–100 μg/mL. Monosaccharide standards including L-guluronic acid (GulUA), D-mannuronic acid (ManUA), D-mannose (Man), D-glucosamine (GlcN), D-ribose (Rib), L-rhamnose (Rham), D-glucuronic acid (GlcUA), D-galacturonic acid (GalUA), D-galactosamine (GalN), D-glucose (Glc), D-galactose (Gal), D-xylose (Xyl), L-arabinose (Ara), and L-fucose (Fuc) were processed similarly for identification and quantification.

#### 2.3.3 FT-IR spectroscopy

Fourier Transform Infrared (FT-IR) spectra were recorded on a Thermo Fisher Scientific FT-IR spectrometer using the KBr pellet method. Approximately 2.0 mg of dried polysaccharide sample was mixed with 200 mg of spectroscopic grade KBr and pressed into transparent pellets under hydraulic pressure. Spectra were acquired in the frequency range of 4,000–400 cm^−1^ with a resolution of 4 cm^−1^ and 32 scans per sample.

#### 2.3.4 UV–vis spectroscopy

Ultraviolet–Visible (UV–Vis) spectra were obtained using a Shimadzu UV-2700 spectrophotometer. Polysaccharide samples were dissolved in distilled water at a concentration of 1.0 mg/mL and scanned from 200 to 800 nm with a scanning interval of 1 nm. The spectra were analyzed for the presence of protein (absorption at 260–280 nm) and other chromophores to assess sample purity.

#### 2.3.5 Zeta potential and particle size analysis

Zeta potential and particle size distribution were measured using a Malvern Zetasizer Nano ZS90 instrument. Polysaccharide solutions (1 mg/mL) were prepared in PBS and filtered through 0.45 μm membranes before analysis. For zeta potential measurements, samples were placed in folded capillary cells and measured at 25 °C with an equilibration time of 120 s. Particle size analysis was performed using dynamic light scattering at 25 °C. Each measurement was performed in triplicate and results were expressed as mean ± standard deviation.

#### 2.3.6 NMR spectroscopy

^1^H NMR spectra were recorded on a Bruker Avance 600 MHz spectrometer at 25 °C. Approximately 30 mg of each polysaccharide sample was dissolved in 0.5 mL of deuterium oxide (D₂O) and freeze-dried twice to exchangeable protons. The fully deuterated samples were then redissolved in 0.5 mL of D₂O for analysis. Spectra were acquired with a spectral width of 12 ppm, acquisition time of 2.7 s, relaxation delay of 1.0 s, and 64 scans.

#### 2.3.7 Antioxidant studies

The free radical scavenging capacities of the polysaccharide samples were evaluated using three established biochemical assays (16). For the 2,2-Diphenyl-1-picrylhydrazyl (DPPH) radical assay, test solutions at varying concentrations were mixed with 0.1 mM DPPH methanolic solution and kept in darkness for 30 min. Absorbance readings were taken at 517 nm, with L-ascorbic acid serving as reference standard. The hydroxyl radical scavenging activity was measured using the Fe^2+^/H₂O₂ system, where sample solutions reacted with phenanthroline, ferrous sulfate, and hydrogen peroxide in PBS. After incubation at 37 °C for 1 h, the absorbance was recorded at 510 nm. Superoxide anion radical scavenging capacity was determined by the pyrogallol autoxidation method. Sample solutions in Tris–HCl buffer (pH 8.2) were mixed with pyrogallol solution, and the absorbance change was monitored at 325 nm for 5 min. All experiments were conducted in triplicate, and radical scavenging rates were calculated using the standard Equation 1:


$$\text{Scavenging rate}(\%)=\frac{A_{c}-A_{s}}{A_{c}}\times 100\%\;\;\;$$ (1)

where *Ac* represents the absorbance of the control group, and *As* represents the absorbance of the sample group. The IC₅₀ values were determined from concentration-response curves.

### 2.4 *In vitro* antidiabetic activity evaluation

The inhibitory activity against *α*-glucosidase was determined according to the chromogenic substrate method with modifications (17). The reaction system comprised 50 μL of sample solution at various concentrations, 50 μL PBS, and 50 μL α-glucosidase solution (0.5 U/mL) dissolved in the same buffer. After pre-incubation at 37 °C for 15 min, 50 μL of 5 mM p-nitrophenyl-*α*-D-glucopyranoside (pNPG) substrate solution was added to initiate the enzymatic reaction. The mixture was further incubated at 37 °C for 30 min, and the reaction was terminated by adding 100 μL of 0.2 M sodium carbonate solution. The amount of p-nitrophenol released was quantified by measuring the absorbance at 405 nm using a microplate reader. The inhibition of *α*-amylase was evaluated using the 3,5-dinitrosalicylic acid (DNSA) method (18). Briefly, 100 μL of sample solution at different concentrations was mixed with 100 μL of α-amylase solution (1 U/mL) in PBS. The mixture was incubated at 37 °C for 15 min, followed by addition of 100 μL of 1% soluble starch solution as substrate. After 15 min of reaction at 37 °C, the enzymatic hydrolysis was stopped by adding 200 μL of DNSA reagent. The reaction tubes were then heated in boiling water bath for 10 min, cooled to room temperature, and diluted with 2 mL of distilled water. The amount of reducing sugars released was determined by measuring the absorbance at 540 nm. Acarbose was used as positive control. The inhibition percentage was calculated as the follow Equation 2:


$$\text{Inhibition}(\%)=\frac{1-A_{s}-A_{b}}{A_{c}}\times 100\%\;\;\;$$ (2)

where *As* represents the absorbance with both enzyme and sample, *Ab* represents the absorbance with sample but without enzyme, and *Ac* represents the absorbance with enzyme but without sample.

For both assays, all experiments were performed in triplicate, and IC₅₀ values were determined by nonlinear regression analysis of the concentration-response curves.

### 2.5 *In vivo* antidiabetic activity evaluation

#### 2.5.1 Experimental animals and diabetes induction

All animal experiments were conducted in accordance with the National Institutes of Health Guide for the Care and Use of Laboratory Animals and were approved by the Ethics Committee of Hubei Engineering University (Approval No. HEUSK202402111). Euthanasia was performed humanely at the end of the study period. All surgical instruments and contact surfaces were sterilized with 75% ethanol or UV irradiation prior to use. Anesthesia was induced by intraperitoneal injection of sodium pentobarbital at 50 mg/kg for painless dissection, and euthanasia via an excessive dose of intraperitoneal injection of sodium pentobarbital at 150 mg/kg.

Male C57BL/6 mice (6–8 weeks old, ~20 g) were housed under controlled conditions (22 ± 2 °C, 55 ± 10% humidity) with a 12 h light/dark cycle. To induce type 2 diabetes (T2DM), all mice except those in the normal control group were fed a high-fat diet (HFD) for 28 consecutive days to establish insulin resistance, followed by a single intraperitoneal injection of freshly prepared STZ (35 mg/kg body weight) in 0.1 M cold citrate buffer (pH 4.5) after an overnight fast, that is a well-recognized protocol for T2DM model establishment (19). The normal control group remained on a standard diet and received an equivalent injection of citrate buffer only. 72 h after STZ administration, mice with fasting blood glucose levels ≥ 11.1 mmol/L were considered diabetic and enrolled in the subsequent intervention study.

#### 2.5.2 Experimental design and treatment protocol

The mice were randomly assigned into six experimental groups (n = 8 per group): Group I served as the normal control and received citrate buffer only; Group II constituted the diabetic model (DM) group and was administered saline; Group III represented the positive control group (MET) and received metformin at 200 mg/kg/day; while Groups IV-VI comprised the polysaccharide treatment groups and received OCP-1, OCP-2, and OCP-3 at 200 mg/kg/day, respectively. This dose was selected based on the safe and effective range of 100–300 mg/kg/day for antidiabetic plant polysaccharides reported in relevant literature (20), and this dose is consistent with the dose of metformin used as the positive control in this study. All treatments were administered orally by gavage for 4 weeks. Body weight and fasting blood glucose levels were measured weekly. Blood glucose was monitored using a glucometer from tail vein blood after 12 h fasting.

#### 2.5.3 Biochemical parameters analysis

At the end of the treatment period, an oral glucose tolerance test (OGTT) was performed after overnight fasting. Blood samples were collected from the tail vein at 0, 30, 60, 90 and 120 min after oral administration of a glucose solution (2 g/kg). The area under the curve (AUC) for glucose tolerance was calculated using the trapezoidal rule. Serum insulin levels were determined using a commercial mouse insulin ELISA kit according to the manufacturer’s instructions.

#### 2.5.4 Organ index and histopathological examination

After 4 weeks of treatment, mice were anesthetized and sacrificed. The liver, kidneys, and pancreas were immediately excised, weighed, and the organ-to-body weight ratios were calculated. Tissue samples were fixed in 10% neutral buffered formalin, processed through graded alcohols and xylene, embedded in paraffin, sectioned at 5 μm thickness, and stained with hematoxylin and eosin (H&E) for histological evaluation under light microscopy. Histological changes were assessed using a semi-quantitative scoring system for organ damage severity, applied in a blinded manner by two independent pathologists based on established criteria (21).

#### 2.5.5 Oxidative stress markers measurement

Liver tissues were homogenized in ice-cold PBS and centrifuged at 12,000 × g for 15 min at 4 °C. The supernatants were used for determination of superoxide dismutase (SOD) activity using the xanthine oxidase method, malondialdehyde (MDA) content by thiobarbituric acid reaction, catalase (CAT) activity by measuring the decomposition of hydrogen peroxide, and glutathione peroxidase (GSH-Px) activity via a commercial kit based on the NADPH oxidation method. All assays were performed using commercial kits following the manufacturers’ protocols.

### 2.6 Safety evaluation

To evaluate the safety profile of the polysaccharide components, a comparative analysis was conducted focusing exclusively between the normal control group and the polysaccharide-treated group (OCP-3). At the experimental endpoint, blood samples were collected for comprehensive safety assessment. Hematological parameters including white blood cell count (WBC), red blood cell count (RBC), hemoglobin concentration (HGB), hematocrit (HCT), and platelet count (PLT) were determined using an automated hematology analyzer. Hepatic and renal function markers comprising alanine aminotransferase (ALT), aspartate aminotransferase (AST), alkaline phosphatase (ALP), albumin (ALB), and creatinine (CR) were measured in serum samples using standardized enzymatic methods and commercial diagnostic kits.

### 2.7 Statistical analysis

All experimental data are expressed as mean values accompanied by standard deviations. For comparative purposes, intergroup differences were assessed using Student’s t-test when comparing two experimental conditions, while one-way analysis of variance (ANOVA) was implemented for evaluations involving multiple experimental groups, followed by Tukey’s *post hoc* test for multiple pairwise comparisons. Statistical thresholds were established at *p < 0.05, **p < 0.01, and ***p < 0.001 to denote varying degrees of significance, with “ns” indicating non-significant differences. All statistical computations were performed utilizing the SPSS statistical package (Release 13.0.0, SPSS Inc., Chicago, IL, USA).

## 3 Results

### 3.1 Chemical composition of OCPs

The chemical profiles of three polysaccharide fractions isolated from *O. cumana* through sequential extraction are summarized in Table 1. The extraction yields showed a remarkable gradation, with alkaline-extracted OCP-3 exhibiting the highest yield (5.01%), significantly surpassing OCP-1 (1.32%) and OCP-2 (0.75%). This trend demonstrates the superior efficiency of alkaline conditions in liberating polysaccharides tightly bound within the cell wall matrix (22). Concurrently, total sugar content progressively increased from OCP-1 (65.6%) to OCP-3 (88.1%), indicating enhanced polysaccharide purity through successive extraction stages. The substantial reduction in protein content from 2.81% (OCP-1) to 0.83% (OCP-3), along with decreased phenolic (0.71 to 0.38%) and flavonoid compounds (4.08 to 3.42%), confirms the effectiveness of the sequential protocol in obtaining polysaccharide-enriched fractions with minimal co-extracted impurities.

**Table 1:** Composition of OCPs and basic chemical characters.

Samples	Yield (%)	Total sugar (%)	Protein (%)	Phenolic (%)	Flavonoid (%)
OCP-1	1.32 ± 0.36^a^	65.6 ± 2.46^a^	2.81 ± 0.14^a^	0.71 ± 0.04^a^	4.08 ± 0.07^a^
OCP-2	0.75 ± 0.12^a^	75.1 ± 8.35^b^	0.55 ± 0.33^b^	0.23 ± 0.02^b^	3.73 ± 0.04^b^
OCP-3	5.01 ± 0.51^b^	88.1 ± 4.12^c^	0.83 ± 0.79^c^	0.38 ± 0.05^c^	3.42 ± 0.05^b^

The different superscript letters indicate a significant difference (*p* < 0.05).

HPGPC analysis revealed distinctive molecular weight patterns among the fractions (Figure 1B). Interestingly, OCP-2 displayed the highest Mw (94,871 Da), suggesting that high-temperature water extraction effectively liberates larger, possibly more entangled polysaccharide polymers from the plant cell wall. In contrast, OCP-1 showed a moderately high Mw (86,100 Da), while OCP-3 exhibited the lowest value (66,273 Da). This significant molecular weight reduction in OCP-3 unequivocally results from *β*-elimination degradation of glycosidic bonds under alkaline conditions, leading to polymer chain cleavage and production of lower molecular weight fragments (23).

### 3.2 Structural properties of OCPs

Monosaccharide profiles revealed fundamental structural differences that correlate with extraction methodology (Figure 1C). OCP-1 and OCP-2 exhibited similar patterns characteristic of heteroxylans or arabinogalactans. OCP-1 was composed of Glc (32.7 mol%), Gal (20.0 mol%), Ara (18.1 mol%), Rha (5.8 mol%), GalA (8.0 mol%), GlcUA (5.2 mol%), Xyl (4.3 mol%), Man (1.5 mol%), Rib (1.8 mol%), and minor components (<1.1 mol%). Similarly, OCP-2 contained Glc (35.9 mol%), Gal (19.9 mol%), Ara (17.1 mol%), GlcUA (6.9 mol%), Rha (5.8 mol%), GalA (5.5 mol%), Xyl (3.6 mol%), and other sugars (<1.3 mol%). The notable presence of GalA and GlcUA contributes to their anionic character. In contrast, OCP-3 displayed a highly specialized composition dominated by Glc (50.7 mol%) and Rha (32.8 mol%), representing a classic signature of rhamnogalacturonan-I (RG-I) pectic polysaccharides (24), with significantly reduced levels of Gal (4.8 mol%), Ara (3.5 mol%), GalA (4.5 mol%), Xyl (1.9 mol%), and other minor constituents (<0.4 mol%). The dramatic increase in Rha content, coupled with pronounced decreases in Ara and Gal, indicates that alkaline conditions effectively solubilize specific pectic polysaccharides inaccessible to aqueous solvents.

Methylation analysis provided detailed insights into glycosidic linkage patterns and structural architectures (Table 2). OCP-1 and OCP-2 featured backbones rich in →4)-*β*-Glcp and →4)-β-Galp residues, with significant branching indicated by →3,6)-Galp linkages. The abundant arabinan side chains, evidenced by high proportions of terminal-Ara and →5)-Ara linkages, explain the substantial arabinose content observed in monosaccharide analysis. Conversely, OCP-3 exhibited a linkage profile diagnostic of RG-I pectin, characterized by predominant →2)-Rha and →2,4)-Rha linkages forming the characteristic backbone disaccharide unit. The notably low levels of terminal Ara and Gal linkages suggest relatively short or simple neutral side chains. The concurrent high abundance of →4)-β-Glcp linkages implies co-extraction of cellulose-like or Xyl-derived glucans tightly associated with the RG-I domain under alkaline conditions.

**Table 2:** Dominant glycosidic linkages in OCPs revealed by methylation analysis.

Methylated sugar derivative	Linkage type	Molar ratio (mol%)		
		OCP-1	OCP-2	OCP-3
2,3,4,6-tetra-O-methyl- glucitol	Terminal-Glcp	5.2	6.0	3.0
2,3,6-tri-O-methyl- glucitol	→4)-Glcp-(1→	15.0	18.0	25.0
2,4,6-tri-O-methyl- glucitol	→3)-Glcp-(1→	3.0	3.0	1.0
2,4-di-O-methyl- glucitol	→3,6)-Glcp-(1→	2.8	3.0	7.0
2,3,4,6-tetra-O-methyl-galactitol	Terminal-Galp	6.5	6.5	1.0
2,3,6-tri-O-methyl- galactitol	→4)-Galp-(1→	8.0	7.5	-
2,4-di-O-methyl-galactitol	→3,6)-Galp-(1→	6.0	5.5	1.5
2,3,5-tri-O-methyl-arabitol	Terminal-Ara	8.5	8.0	1.5
2,3,4-tri-O-methyl-arabitol	→5)-Ara-(1→	10.0	9.0	1.0
3,4-di-O-methyl-rhamnitol	→2)-Rha-(1→	2.0	2.0	15.0
3-O-methyl-rhamnitol	→2,4)-Rha-(1→	1.5	1.5	10.0
2,3-di-O-methyl-galactitol	→4,6)-GalA-(1→	4.0	3.0	2.5

**Figure 2:**
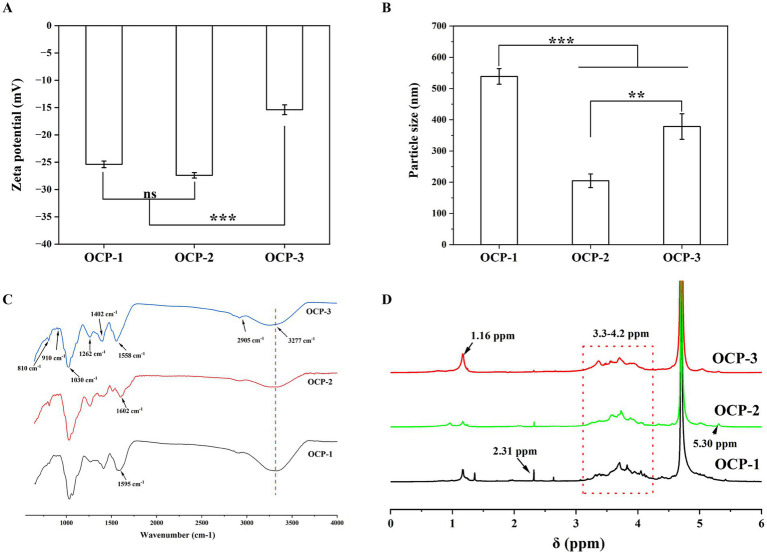
Physicochemical characterization of OCPs. **(A)** Zeta potential values. **(B)** Particle size distribution. **(C)** FT-IR spectra. **(D)** ^1^H NMR spectra.

The surface charge and aggregation state of the OCP fractions, as determined by zeta potential and particle size measurements (Figures 2A,B), revealed distinct physicochemical behaviors. OCP-1 and OCP-2 exhibited relatively high negative zeta potentials (−25.4 mV and −27.4 mV, respectively), consistent with their significant content of anionic uronic acids identified in the monosaccharide analysis. This strong electrostatic repulsion contributed to the notably small particle size of OCP-2 (204 nm). In contrast, OCP-1 formed larger aggregates (538 nm), likely due to chain entanglement of its high-molecular-weight polymers overcoming the moderate electrostatic stabilization. OCP-3 presented a markedly different profile, with a substantially lower zeta potential (−15.4 mV), aligning with its reduced uronic acid content and dominance of neutral sugars. Despite possessing the lowest molecular weight, its weakened electrostatic repulsion facilitated the re-association of degraded polymer chains, resulting in an intermediate particle size (378 nm) (25).

FT-IR spectra confirmed the polysaccharidic nature of all fractions while revealing distinct structural features (Figure 2C). Common characteristic absorptions included O-H stretching (3,277 cm^−1^), C-H stretching (2,905 cm^−1^), and glycosidic bond vibrations (1,030 cm^−1^). The critical distinctions emerged in the carboxylate region (1500–1,650 cm^−1^), where OCP-3 showed a sharp peak at 1558 cm^−1^ indicative of ionized carboxylate groups, aligning with its identity as RG-I pectin where GalA residues are deprotonated under alkaline extraction (26). OCP-2 displayed absorption at 1602 cm^−1^ characteristic of protonated carboxylic acids, consistent with glucuronic acid content in its heteroxylan/arabinogalactan structure preserved under milder extraction. OCP-1 exhibited a broad feature around 1,595 cm^−1^, suggesting a mixture of protonated and ionized species from co-extracted acidic polysaccharides.

^1^H NMR spectra provided additional evidence for structural differences (Figure 2D). The most distinctive feature in OCP-3 was the dominant signal at 1.16 ppm, assigned to methyl protons of *α*-L-Rha, consistent with its RG-I identity where Rha constituted approximately 33% of composition. The anomeric proton signal at 5.03 ppm further confirmed α-linked glycosides typical for RG-I structures (27). OCP-2 showed a different pattern with an anomeric proton at 5.30 ppm, indicating α-linked residues in its arabinogalactan structure. OCP-1 presented the most complex profile with signals at 1.36 ppm and 2.31 ppm potentially from acetyl groups or phenolic compounds, and limited α-linkages (5.41 ppm), suggesting predominantly *β*-configurations (28).

In conclusion, the sequential extraction process successfully fractionates OCPs based on their structural identity and integration within the cell wall matrix. The methodology isolates branched arabinogalactans (OCP-1 and OCP-2) in the initial aqueous stages, culminating in the liberation of a specialized RG-I-glucan structural complex (OCP-3) under alkaline conditions.

### 3.3 *In vitro* antioxidant activities of OCPs

**Figure 3:**
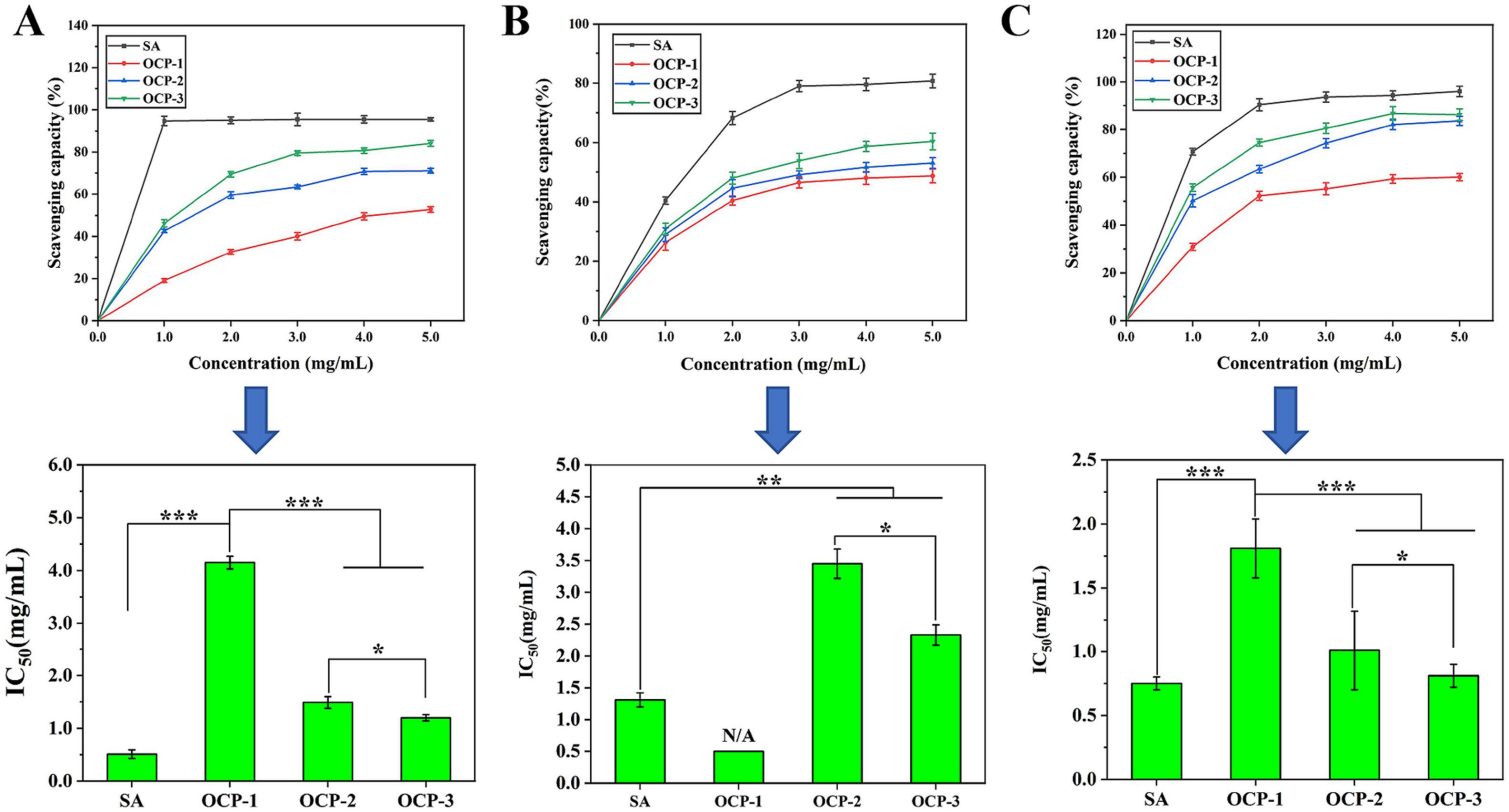
Antioxidant activities of OCPs. **(A)** DPPH radical scavenging activity. **(B)** Hydroxyl radical scavenging capacity. **(C)** Superoxide anion radical scavenging effect. Data are presented as mean ± SD (*n* = 3). Ascorbic acid (SA) was used as positive control. Statistical significance compared to control group is indicated in the text with IC_50_₅₀ values.

Given that oxidative stress is a well-established contributor to insulin resistance and pancreatic β-cell dysfunction, the assessment of antioxidant capacity provides critical mechanistic insights into the potential hypoglycemic effects of OCPs (29). To better reflect the antioxidant potential of OCPs, SA was used as a positive control in all three radical scavenging assays, with IC_50_ values of 0.75 mg/mL (DPPH radical), 1.89 mg/mL (hydroxyl radical), and 0.75 mg/mL (superoxide anion radical), respectively. Our comprehensive evaluation revealed a consistent structure–activity relationship across three distinct free radical models (Figure 3), with OCP-3 demonstrating superior scavenging capacity followed by OCP-2 and OCP-1. In the DPPH radical assay, OCP-3 exhibited the strongest activity (IC₅₀ = 1.20 mg/mL), significantly outperforming OCP-2 (IC₅₀ = 1.49 mg/mL) and OCP-1 (IC₅₀ = 4.15 mg/mL). Concentration-dependent analysis further confirmed this trend, with OCP-3 achieving 80.0% scavenging efficiency at 3 mg/mL, markedly higher than OCP-1 (40.0%) and OCP-2 (64.0%). The hydroxyl radical scavenging assay yielded similar results, with OCP-3 showing the lowest IC₅₀ value (2.33 mg/mL) and reaching 60.4% scavenging at 5 mg/mL, compared to 53.0% for OCP-2 and 48.7% for OCP-1. Most notably, in superoxide anion radical scavenging, particularly relevant to diabetes pathology, OCP-3 demonstrated exceptional potency (IC₅₀ = 0.81 mg/mL), achieving 86.7% inhibition at 4 mg/mL and approaching the efficacy of the SA control (IC₅₀ = 0.75 mg/mL). Overall, compared with SA, OCP-3 showed comparable superoxide anion radical scavenging activity and moderate DPPH and hydroxyl radical scavenging activities, indicating its potential as a natural antioxidant alternative to synthetic standards.

It should be acknowledged that *in vitro* antioxidant assays are conducted under simplified conditions that differ from the complex physiological environment *in vivo*. Thus, the antioxidant capacity observed *in vitro* may not fully reflect the actual efficacy *in vivo*, and the *in vivo* antioxidant effects should be primarily evaluated based on the changes in endogenous oxidative stress markers.

### 3.4 *In vitro* antidiabetic activities

**Figure 4:**
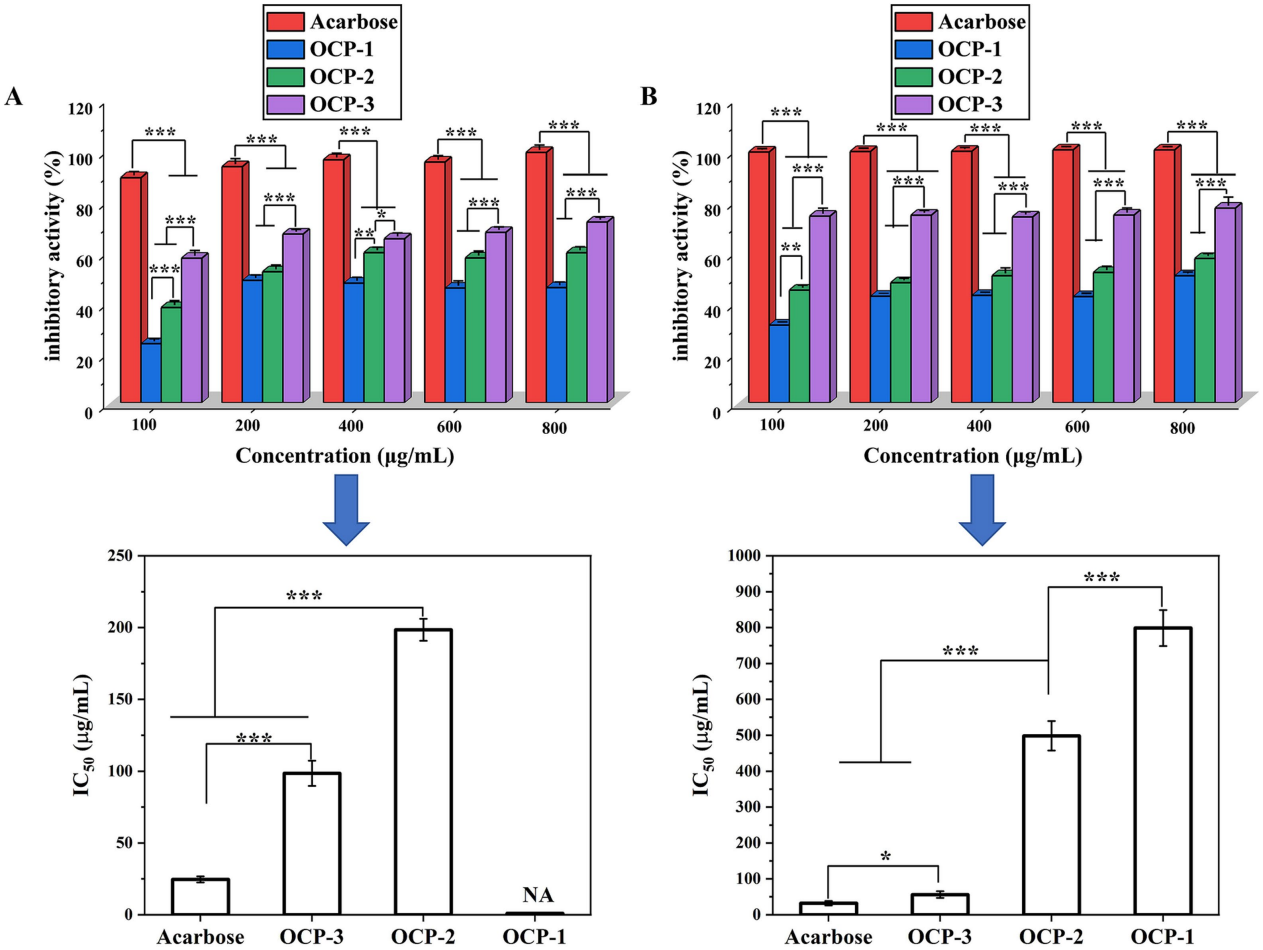
Inhibitory effects of OCPs on carbohydrate-digesting enzymes at different concentrations. **(A)** *α*-Amylase inhibition activity. **(B)** α-Glucosidase inhibition activity. Data are presented as mean ± SD (*n* = 3). Acarbose was used as positive control. Statistical significance compared to control group is indicated in the text with IC values.

The suppression of carbohydrate-digesting enzymes, specifically *α*-amylase and α-glucosidase, offers a targeted therapeutic approach for moderating postprandial hyperglycemia—a key factor in type 2 diabetes management (30). By delaying the breakdown of starch and disaccharides into absorbable monosaccharides, this strategy effectively slows glucose absorption and attenuates post-meal blood glucose excursions. The inhibitory effects of OCPs on carbohydrate-digesting enzymes revealed distinct structure–activity relationships (Figure 4). Against *α*-amylase, OCP-3 demonstrated the strongest inhibition (IC₅₀ = 98.5 μg/mL), significantly surpassing OCP-2 (IC₅₀ = 198.5 μg/mL), while OCP-1 showed negligible activity. More notably, in α-glucosidase inhibition assays, OCP-3 exhibited remarkable potency (IC₅₀ = 56.1 μg/mL), approaching the efficacy of acarbose and substantially outperforming OCP-2 (IC₅₀ = 498.2 μg/mL) and OCP-1 (IC₅₀ = 799 μg/mL). The superior enzyme inhibitory capacity of OCP-3 is consistent with its unique structural characteristics. The abundance of Rha residues (32.8 mol%) and associated GalUA moieties in OCP-3 may enhance competitive binding to the enzymes’ catalytic domains. Furthermore, the presence of co-extracted glucan chains (50.7 mol% Glc) could provide additional binding sites through hydrogen bonding and hydrophobic interactions. OCP-3’s particularly strong inhibition of α-glucosidase indicates its potential to specifically target the final step of carbohydrate digestion, thereby moderating postprandial glucose absorption more effectively than conventional inhibitors.

### 3.5 *In vivo* antidiabetic effects of OCPs

**Figure 5:**
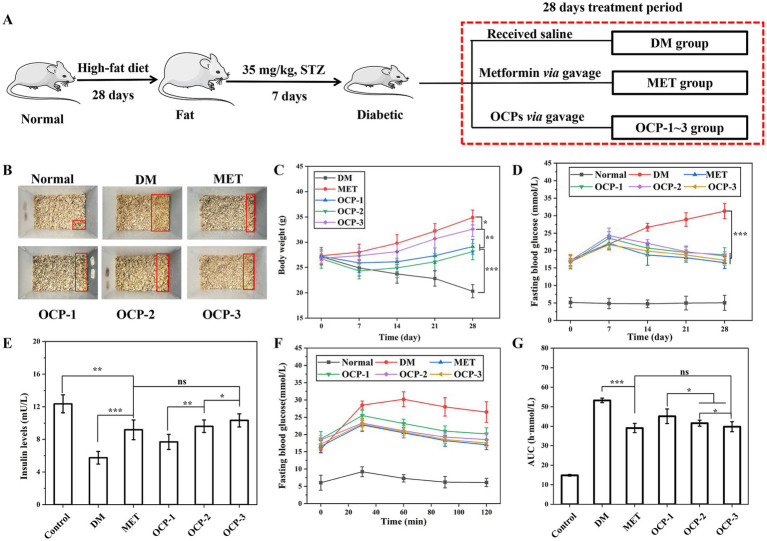
*In vivo* antidiabetic effects of OCPs in STZ-induced diabetic mice. **(A)** Schematic representation of the experimental design with six groups: Normal control, DM model, MET, and OCPs-treated groups. **(B)** Urine output ratio in cage bedding. **(C)** Body weight changes monitored every 4 days over 28 days. **(D)** Fasting blood glucose levels measured every 4 days during the treatment period. **(E)** Serum insulin levels after 28 days of treatment. **(F)** Postprandial blood glucose profiles during OGTT measured at 20 min intervals. **(G)** Corresponding area AUC values for OGTT. Data are presented as mean ± SD (*n* = 8). Statistical significance: *p < 0.05, **p < 0.01, *p < 0.001 vs. DM group.

The comprehensive *in vivo* assessment revealed substantial improvements in diabetic parameters following OCPs administration, with OCP-3 demonstrating efficacy approaching that of the metformin control (Figure 5). Analysis of polyuria symptoms indicated notable recovery across treatment groups (Figure 5B). The DM group exhibited extensive urine coverage (42.5% of bedding area), whereas OCP-3 treatment substantially reduced this parameter to 15.2%, closely mirroring the metformin group (13.8%). Intermediate effects were observed in OCP-1 (28.3%) and OCP-2 (22.7%) groups, while normal animals maintained minimal urine output (5.2%). Body weight trajectories revealed distinct recovery patterns among groups (Figure 5C). Diabetic controls experienced progressive wasting, declining from 27.1 g to 20.3 g over 28 days. Metformin treatment supported continuous weight gain (27.3 g to 34.9 g), while OCP-3 administration promoted the most robust weight recovery among polysaccharide groups (26.8 g to 32.6 g), significantly surpassing both OCP-1 (29.1 g) and OCP-2 (28.1 g) by study termination. Glycemic control profiles demonstrated treatment-dependent regulation (Figure 5D). The DM group developed progressive hyperglycemia (16.7 mM to 31.3 mM), whereas MET-maintained glucose at 16.5 mM. OCP-3 treatment yielded superior glucose regulation (17.2 mM) compared to OCP-1 (18.7 mM) and OCP-2 (18.4 mM), with normal animals maintaining euglycemia (5.0 mM). Post-treatment insulin measurements revealed critical restoration of endocrine function (Figure 5E). The DM group showed severe insulin deficiency (5.74 mU/L) compared to normal controls (12.37 mU/L). Remarkably, OCP-3 treatment achieved the highest insulin levels among interventions (10.34 mU/L), significantly exceeding OCP-1 (7.68 mU/L) and OCP-2 (9.62 mU/L), and slightly surpassing MET (9.17 mU/L). OGTT further validated OCP-3’s therapeutic potential (Figures 5F,G). During OGTT, OCP-3 treated animals reached 17.5 mM at 120 min, significantly lower than OCP-1 (20.2 mM, *p* < 0.05) and OCP-2 (19.1 mM, p < 0.05), and comparable to MET (17.0 mM). Area under curve analysis confirmed these findings, with OCP-3 (39.83 h·mmol/L) demonstrating significantly improved glucose tolerance versus OCP-1 (45.15 h·mmol/L) and OCP-2 (41.60 h·mmol/L), while approaching MET efficacy (39.13 h·mmol/L).

**Figure 6:**
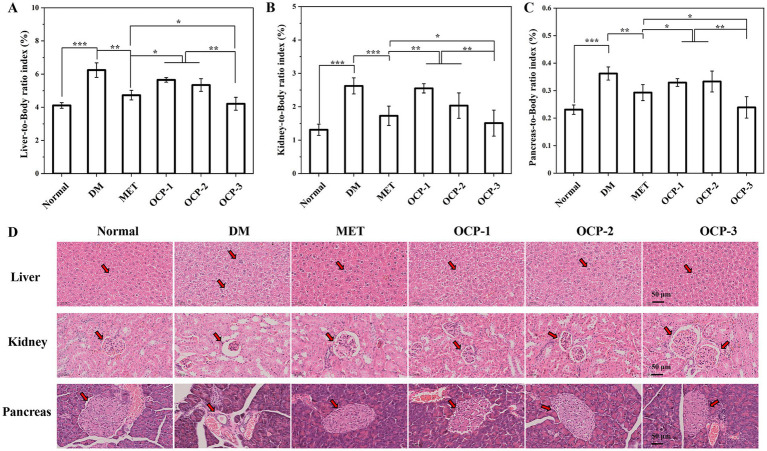
Organ weight indices and histopathological examination. **(A)** Liver-to-body weight ratio. **(B)** Kidney-to-body weight ratio. **(C)** Pancreatic-to-body weight ratio. **(D)** Representative H&E-stained sections of liver, kidney, and pancreatic tissues (scale bar: 50 μm) from one representative animal per group (*n* = 8 per group, all showed similar patterns).

Evaluation of organ coefficients and tissue morphology revealed substantial protection by OCPs against diabetes-induced organ damage, as quantified in Figure 6. The DM group developed significant hepatomegaly with liver-to-body weight ratio reaching 6.24%, representing a 51.8% increase over normal controls (4.11%). OCP-3 treatment effectively normalized this parameter to 4.21%, demonstrating superior hepatoprotection compared to OCP-1 (5.65%) and OCP-2 (5.34%), and approaching metformin efficacy (4.73%). Renal hypertrophy, displayed in Figure 6B, was markedly evident in the DM group (2.62%), showing a 100.3% increase versus normal values (1.31%). OCP-3 treatment substantially reduced kidney enlargement to 1.51%, significantly outperforming OCP-1 (2.55%) and OCP-2 (2.03%), and nearing MET’s effect (1.73%). Pancreatic coefficients, presented in Figure 6C, followed a similar pattern, with OCP-3 (0.24%) effectively reversing diabetes-induced alterations (DM: 0.36%) and demonstrating protection comparable to MET (0.29%). Histopathological examination, illustrated in Figure 6D, provided direct morphological evidence of these protective effects. Hepatic tissues from OCP-3 treated animals showed minimal lipid accumulation and preserved hepatocyte architecture, comparable to metformin treatment. Kidney sections revealed that OCP-3 effectively prevented glomerular atrophy and maintained normal glomerular structure, outperforming other OCPs. Most notably, pancreatic histology demonstrated that OCP-3 preserved islet morphology and cellular organization, showing superior protection against diabetes-induced islet atrophy. The comprehensive organ protection by OCP-3, particularly its ability to concurrently preserve hepatic, kidney, and pancreatic integrity, aligns with its demonstrated antioxidant and anti-inflammatory properties.

**Figure 7:**
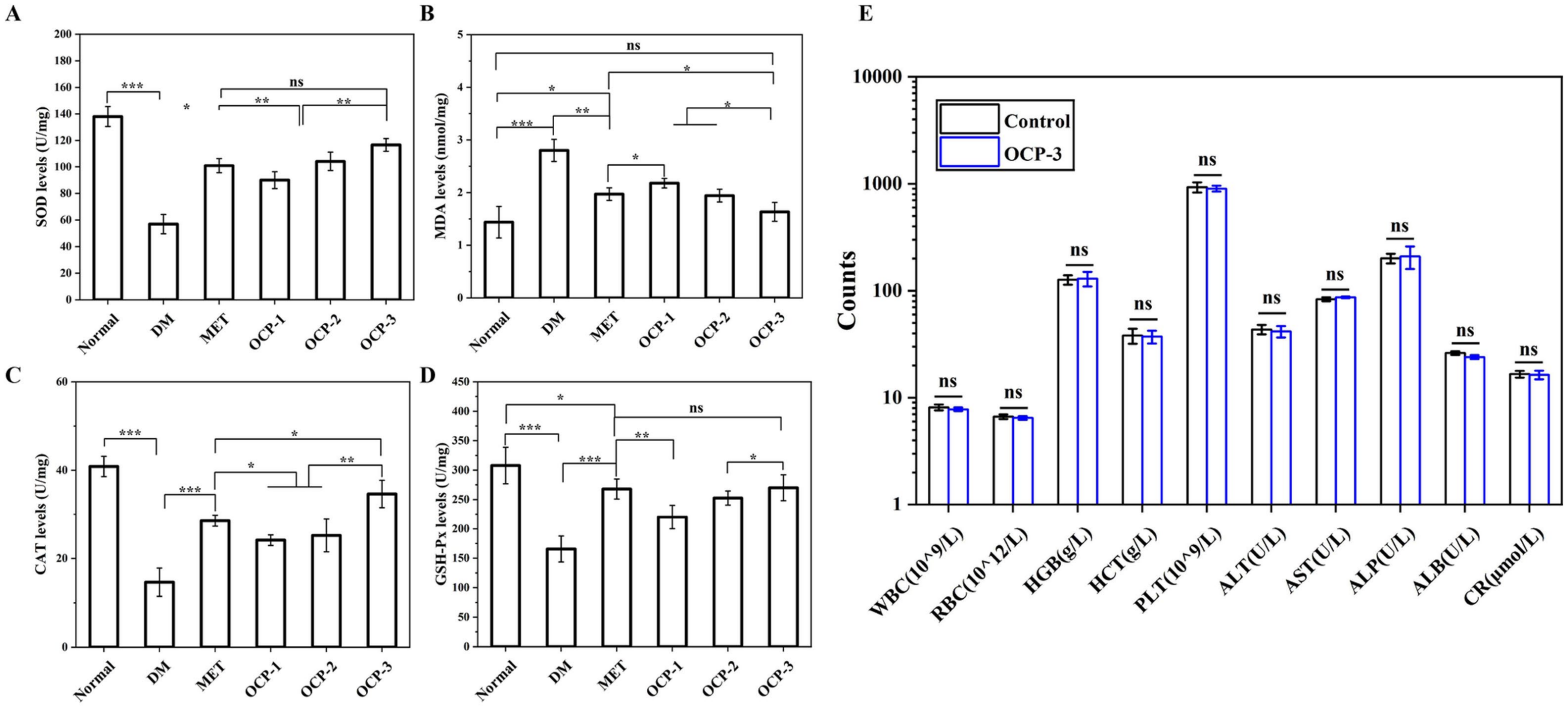
Effects of OCPs on oxidative stress markers and safety evaluation. **(A)** SOD activity. **(B)** MDA content. **(C)** CAT activity. **(D)** GSH-Px activity. **(E)** Serum biochemical analysis for safety assessment (data presented in Table 3).

The systematic evaluation of oxidative stress markers demonstrated the potent antioxidant efficacy of OCPs under diabetic conditions, as comprehensively compared in Figures 7A–D. SOD activity was severely compromised in the DM group (56.91 U/mg), representing only 41.2% of normal levels (138.06 U/mg). OCP-3 treatment significantly restored SOD activity to 116.72 U/mg, achieving 84.5% recovery of normal values and substantially outperforming both OCP-1 (89.99 U/mg) and OCP-2 (104.29 U/mg), while approaching the efficacy of MET treatment (100.99 U/mg). Similarly, MDA content showed a marked increase in the DM group (2.80 nmol/mg), indicating enhanced lipid peroxidation. OCP-3 treatment effectively reduced MDA levels to 1.63 nmol/mg, demonstrating superior protection against oxidative damage compared to other OCPs and even surpassing MET (1.97 nmol/mg). The CAT activity results revealed a parallel trend, with OCP-3 (34.65 U/mg) showing the most significant recovery among polysaccharide treatments, reaching 84.8% of normal catalase levels (40.85 U/mg). Furthermore, GSH-Px activity was severely depleted in diabetic animals (165.62 U/mg) compared to normal controls (307.88 U/mg). OCP-3 treatment demonstrated remarkable efficacy in restoring GSH-Px activity to 270.11 U/mg, significantly higher than OCP-1 (220.12 U/mg) and OCP-2 (252.65 U/mg), and comparable to MET treatment (267.99 U/mg).

**Table 3:** Serum biochemical and hematological parameters.

Parameter	Serum biochemical	Control	OCP-3
White blood cell (WBC)	× 10^9^ /L	8.12 ± 0.51	7.79 ± 0.33
Red blood cell (RBC)	× 10^12^ /L	6.64 ± 0.32	6.48 ± 0.25
Hemoglobin (HGB)	g/L	126.60 ± 13.12	129.61 ± 20.51
Hematocrit (HCT)	g/L	38.02 ± 6.11	37.26 ± 5.10
Platelet (PLT)	× 10^9^/L	930.48 ± 101.02	899.57 ± 56.12
Alanine aminotransferase (ALT)	U/L	43.41 ± 4.45	41.64 ± 5.32
Aspartate aminotransferase (AST)	U/L	83.29 ± 3.61	86.84 ± 1.21
Alkaline phosphatase (ALP)	U/L	200.70 ± 21.44	209.25 ± 50.47
Albumin (ALB)	U/L	26.14 ± 1.08	23.97 ± 1.07
Creatinine (CR)	μmol/L	16.63 ± 1.22	16.36 ± 1.55

The restoration of insulin secretion and preservation of pancreatic islet architecture emerged as the most distinctive therapeutic outcome of OCP-3 treatment. While many antidiabetic polysaccharides act primarily by improving insulin sensitivity or inhibiting digestive enzymes, the capacity to directly address the insulin deficiency central to diabetes pathophysiology is a rarer and highly valuable property. The significant recovery of insulin levels in the OCP-3 group, approaching those of normal controls, strongly implies a direct or indirect protective effect on pancreatic *β*-cells (31). We hypothesize that the potent antioxidant activity of OCP-3, as demonstrated in both *in vitro* and *in vivo* oxidative stress markers, plays a crucial role in mitigating STZ-induced and hyperglycemia-aggravated oxidative damage in β-cells, thereby reducing apoptosis and preserving their function. This potential to not only manage glycemia but also to target the underlying β-cell failure makes OCP-3 a particularly promising candidate for future development and warrants further investigation into its precise mechanisms of β-cell protection and regeneration.

### 3.6 Safety evaluation

The comprehensive safety assessment revealed that OCP-3 administration for 28 days produced no significant alterations in key hematological parameters (WBC, HGB, RBC, HCT, PLT) or serum biochemical markers (AST, ALT, ALP, ALB, CR) compared to normal controls, establishing its favorable safety profile at biologically effective doses (Figure 7E). The selection of OCP-3 for this detailed safety evaluation was justified by its consistently superior bioactivity profile, including the most potent antioxidant and enzyme inhibitory activities, optimal structural characteristics with the lowest molecular weight and unique RG-I composition, and demonstrated efficacy in improving metabolic parameters and organ protection.

## 4 Discussion

The sequential extraction strategy (water extraction, high-temperature water extraction, and alkaline extraction) adopted in this study achieves selective separation of OCPs based on the differences in the binding strength between polysaccharides and the plant cell wall matrix, successfully isolating three polysaccharide fractions with distinct structural characteristics. Specifically, OCP-1 and OCP-2 obtained by water and high-temperature water extraction are arabinogalactans/heteroxylans, while OCP-3 extracted by alkaline conditions is a rhamnogalacturonan-I (RG-I)-glucan complex. Alkaline extraction shows superior efficiency in liberating cell wall-bound polysaccharides with the highest yield (5.01%) and purity (88.1% total sugar content) of OCP-3, which is attributed to the *β*-elimination reaction breaking glycosidic bonds and dissolving cell wall components, verifying the correlation between extraction conditions and structural characteristics of polysaccharides (32).

The bioactivity of OCPs is closely related to their structural characteristics, showing a clear structure–activity relationship with OCP-3 > OCP-2 > OCP-1. Molecular weight is a key factor affecting bioactivity; OCP-3 has the lowest molecular weight (66,273 Da) due to alkaline degradation, which enhances its water solubility, molecular mobility, and accessibility to biological macromolecules such as enzymes and free radicals (33). Recent studies have confirmed that low molecular weight RG-I polysaccharides exhibit superior antioxidant and hypoglycemic activities compared to their high molecular weight counterparts (34, 35). In terms of monosaccharide composition and glycosidic bond types, OCP-1 and OCP-2 are mainly composed of Glc, Gal, and Ara with *β*-Glcp and β-Galp backbones, while OCP-3 is dominated by Glc (50.7 mol%) and Rha (32.8 mol%) with characteristic RG-I disaccharide repeating units (→2)-Rha and →2,4)-Rha linkages) and glucan chains. The flexible backbone of RG-I, abundant hydroxyl and carboxyl groups, and synergistic effect with glucan chains enable OCP-3 to exhibit optimal bioactivity, while the large steric hindrance from arabinan side chains in OCP-1 and OCP-2 limits their bioactivity, consistent with recent report on RG-I-rich pectic polysaccharides from other medicinal plants (36).

OCP-3 exerts significant antidiabetic effects through multi-dimensional mechanisms. Firstly, it effectively inhibits carbohydrate-digesting enzymes (*α*-amylase, IC₅₀ = 98.5 μg/mL; α-glucosidase, IC₅₀ = 56.1 μg/mL), especially showing high potency against α-glucosidase which targets the final step of carbohydrate digestion, thereby delaying glucose absorption and reducing postprandial blood glucose. Secondly, OCP-3 exhibits strong antioxidant activity, scavenging DPPH, hydroxyl, and superoxide anion radicals efficiently. *In vivo*, it restores the activity of antioxidant enzymes (SOD, CAT, GSH-Px) and reduces MDA content in diabetic mice. This antioxidant-mediated *β*-cell protection may further modulate insulin signaling pathways to improve glucose utilization in peripheral tissues, thereby alleviating oxidative stress-induced pancreatic β-cell damage, restoring insulin secretion (10.34 mU/L), and protecting islet morphology. The preserved islet morphology in OCP-3-treated mice also implies the potential of pancreatic β-cell self-restoration under oxidative stress alleviation by OCP-3, which is consistent with the reported self-repair capacity of β-cells under mild damage conditions (37). Additionally, OCP-3 improves metabolic disorders in diabetic mice, including reducing urine output, promoting weight recovery, and stabilizing fasting blood glucose, while also normalizing liver, kidney, and pancreatic coefficients and protecting organ structures from diabetic damage (38).

OCP-3 has considerable application potential as a natural and safe functional ingredient for developing antidiabetic functional foods therapeutic agents. Derived from *O. cumana*, an agricultural pest that causes significant yield losses in sunflower cultivation worldwide, it features abundant resources and simple extraction processes, exemplifying a waste-to-resource strategy that converts an economically damaging plant into a high-value functional ingredient. However, the study still has limitations, such as insufficient in-depth molecular mechanism research, short *in vivo* experimental cycle, single diabetic model, incomplete fine structure characterization of OCP-3, and lack of preclinical pharmacokinetic studies. As this discovery-phase study focuses on structural characterization and initial efficacy evaluation, detailed molecular mechanism studies (e.g., PI3K/AKT, AMPK pathways) are planned for future investigations.

## Data Availability

The raw data supporting the conclusions of this article will be made available by the authors, without undue reservation.
